# Case Report: Congenital gallbladder agenesis

**DOI:** 10.3389/fmed.2026.1778733

**Published:** 2026-01-30

**Authors:** Bo Gao, Xuefeng Bai, Rui Zhang

**Affiliations:** Department of Hepatobiliary Surgery, Affiliated Hospital of Hebei University, Baoding, China

**Keywords:** atrophic cholecystitis, case report, congenital gallbladder agenesis, liver ultrasonography, MRI

## Abstract

The congenital gallbladder agenesis presents a diagnostic challenge due to its atypical clinical features, often leading to misdiagnosis or delayed diagnosis and ultimately unnecessary surgeries. The role of MRI in this condition cannot be overstated. We report a case where MRI preoperatively diagnosed congenital gallbladder agenesis. Initially, routine physical examination ultrasonography misdiagnosed the patient with a gallbladder full of stones and atrophic cholecystitis, leading the patient to seek surgical treatment and get admitted to the hospital. After admission, further abdominal MRI finally diagnosed congenital gallbladder agenesis, successfully avoiding unnecessary surgery. This case underscores the key clinical lesson of leveraging MRI to prevent unnecessary surgeries.

## Introduction

Congenital gallbladder agenesis (GBA), first reported in 1701,is a relatively rare congenital biliary malformation. The incidence reported in literature ranges from 0.01% to 0.04%, with a male-to-female ratio of approximately 1:2 to 1:4 (1–3). This condition is usually incidentally discovered during anatomical or radiological examinations, and its exact etiology remains incompletely understood, possibly related to genetic and environmental factors during embryonic development (4). While most patients may not have obvious symptoms, some may experience intermittent abdominal pain, dyspepsia, and other symptoms related to biliary dysfunction (5), which increases the possibility of misdiagnosis and even unnecessary exploratory surgery. Therefore, clinicians should consider the possibility of this rare condition when dealing with digestive symptoms such as abdominal pain to avoid misdiagnosis and unnecessary surgical interventions.

We present a case of congenital gallbladder agenesis diagnosed preoperatively by MRI. The patient was initially misdiagnosed with a gallbladder full of stones and atrophic cholecystitis by routine physical examination ultrasonography and was admitted for further surgical treatment. After admission, abdominal CT and MRI were further improved, leading to a final diagnosis of congenital gallbladder agenesis and avoiding unnecessary surgery.

## Case presentation

A 53-years-old female patient, with no prior history of hepatobiliary diseases, was admitted to the hospital due to gallbladder stones with gallbladder atrophy were found during a physical examination 1 week ago. During a health check-up, she was considered as a possibility of gallbladder full of stones with gallbladder atrophy based on a hepatobiliary B-ultrasound examination. Although there are no clinical symptoms, considering that both gallstones and atrophic cholecystitis are risk factors for gallbladder cancer, the patient was advised to be hospitalized for elective laparoscopic cholecystectomy.

**FIGURE 1:**
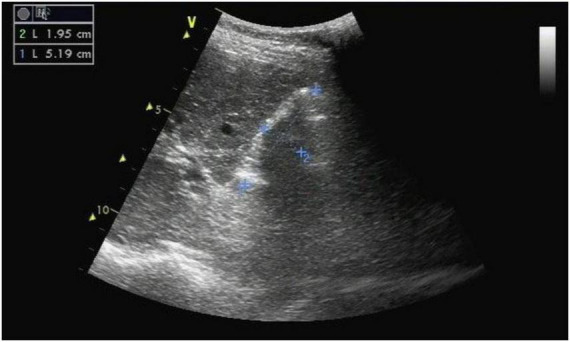
The B-ultrasound revealed that the normal gallbladder structure was not detected in the gallbladder area, and it was replaced by a strong echogenic mass with a range of approximately 5.19 cm × 1.95 cm.

After admission, we rechecked the ultrasound for the patient. Ultrasound revealed that the normal gallbladder structure was not detected in the gallbladder area, and it was replaced by a strong echogenic mass with a range of approximately 5.19 cm × 1.95 cm (Figure 1).

Serum biochemical analyses indicated that liver function indices, renal function, and coagulation parameters were within normal limits. Tumor markers were also within normal ranges (Table 1).

**TABLE 1:** Patient’s laboratory test results.

Laboratory test	Results	Normal range
Alanine aminotransferase (ALT) (U/L)	31	7∼40
Aspartate aminotransferase (AST) (U/L)	28	13∼35
Alkaline phosphatase (ALP) (U/L)	53	50∼135
Gamma-glutamyl transferase (GGT) (U/L)	12	7∼45
Total bilirubin (umol/L)	12.46	≤23.00
Direct bilirubin (umol/L)	4.13	0∼6.80
Indirect bilirubin (umol/L)	8.33	0∼16.20
Cancer antigen 19-9 (CA19-9) (U/mL)	6.10	0∼39.00

A provisional diagnosis of cholecystolithiasis with atrophic cholecystitis was made, and the patient was planned for laparoscopic cholecystectomy after obtaining informed consent.

Abdominal CT suggested: the gallbladder is not clearly visible, and there is mild dilation of intrahepatic and extrahepatic bile ducts.

**FIGURE 2:**
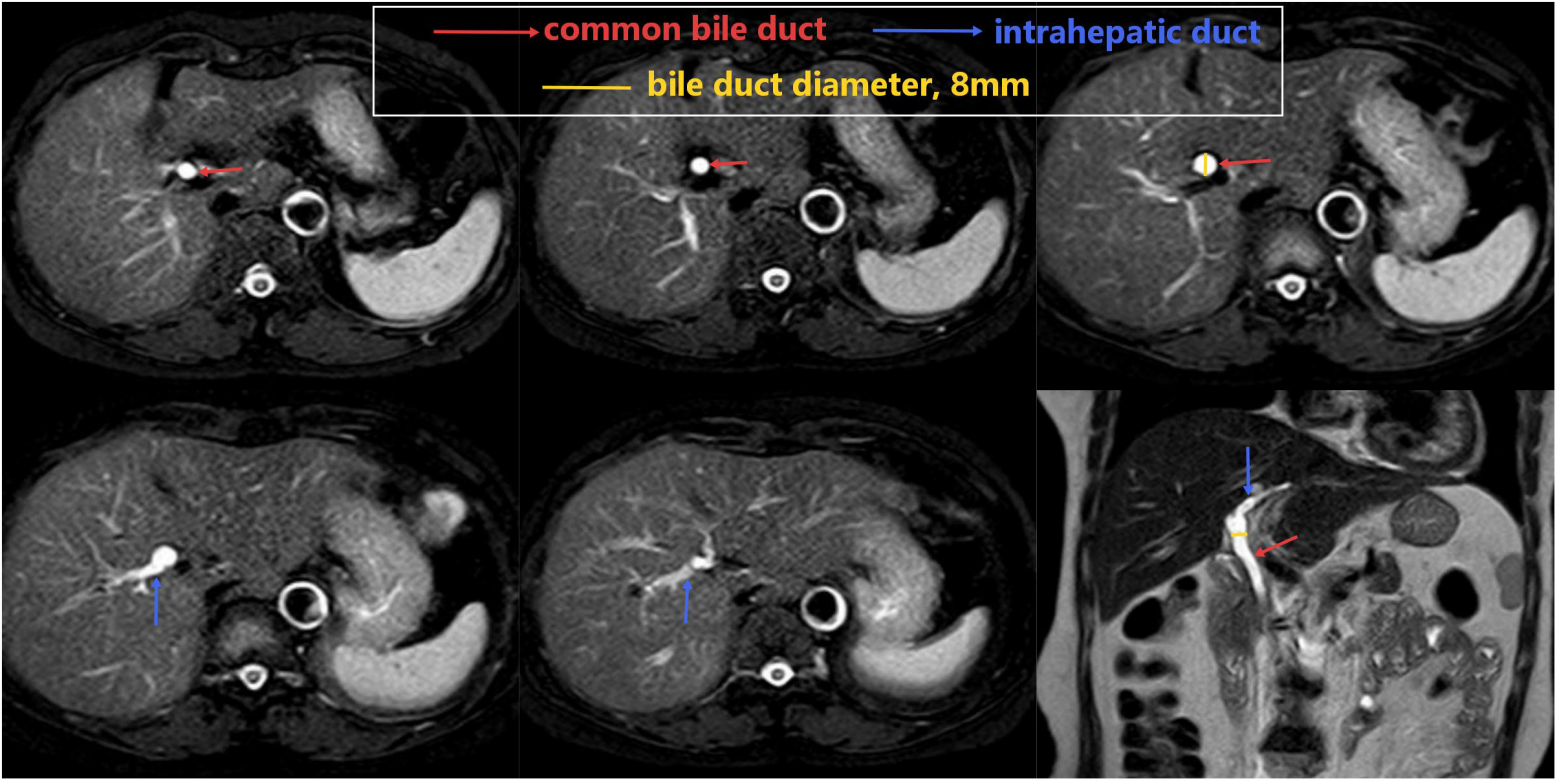
Magnetic resonance imaging (MRI) showed no clear gallbladder structure is visible, and the bile duct diameter is at the upper limit. From the lower end of the bile duct to the intrahepatic bile duct, the continuity of the bile duct wall is visible, and no cystic duct branches are seen.

However, abdominal magnetic resonance imaging (MRI) showed no clear gallbladder structure is visible, and the bile duct diameter is at the upper limit (Figure 2).

Based on the patient’s MRI examination results, the definitive diagnosis was CGA, thereby avoiding unnecessary surgery. At follow-up visits at 1 month, 6 months, 1 year, and 3 years, the patient reported no significant discomfort, and both the patient and her family were satisfied with the definitive diagnosis and the avoidance of unnecessary surgery.

## Discussion

Congenital gallbladder agenesis (CGA) is an extremely rare congenital anomaly characterized by the complete absence of the gallbladder, which is a significant component of the biliary system. This condition can be isolated, but a substantial minority of cases are reported with other congenital abnormalities–most commonly involving the genitourinary, gastrointestinal, and cardiovascular systems, such as ventricular septal defect, imperforate anus, duodenal atresia, malrotation of the gut, pancreas divisum, hypoplasia of the right hepatic lobe, duplication cysts of the hepatic flexure, renal agenesis, undescended testes, and syndactyly (6). The embryological basis for CGA involves a failure of gallbladder development during the early stages of fetal life, specifically between the 4th and 8th weeks of gestation. This developmental failure can be attributed to various factors, including genetic predisposition and environmental influences (4).

Clinically, gallbladder agenesis is categorized into three groups based on presentation: the asymptomatic type (35%), the symptomatic type (50%), and the fatal type (15%–16%) (4). This condition should be distinguished from other conditions that present with a non-visualized gallbladder, such as: chronic cholecystitis with a shrunken/fibrotic gallbladder, Porcelain gallbladder, Post-surgical absence (cholecystectomy), Ectopic gallbladder location. When MRI showed no clear gallbladder structure is visible, the continuity of the bile duct wall is visible, no cystic duct branches are seen, and the patient has no history of cholecystectomy, CGA should be considered first.

While it is generally believed that CGA is not typically inherited, genetic factors may contribute to an increased risk of developing the condition to a certain extent. In particular, the presence of CGA cases in a family may elevate the risk of the offspring being affected. This may be due to specific genetic mutations related to gallbladder development that can interfere with the normal developmental process of the gallbladder.

The clinical significance of CGA lies in its potential to cause various symptoms, although many patients remain asymptomatic throughout their lives. Those who do present with symptoms may experience biliary colic, chronic abdominal pain, or complications related to the biliary system, such as sphincter of Oddi dysfunction (4). The absence of the gallbladder can complicate the diagnosis of biliary diseases, as standard imaging techniques like ultrasound may fail to visualize the anomaly, leading to misdiagnosis or unnecessary surgical interventions. Moreover, CGA may also be associated with other congenital abnormalities, which can further complicate the clinical picture and affect the management strategies employed (7).

In this case, from the lower end of the bile duct to the intrahepatic bile duct, the continuity of the bile duct wall is visible, and no cystic duct branches are seen. Based on the MRI results, the patient was ultimately diagnosed with congenital agenesis of the gallbladder, thereby avoiding unnecessary surgery.

In the future, for patients with suspected atrophic cholecystitis, but standard imaging fails to identify the gallbladder, Non-visualization of the gallbladder on ultrasound/MRI, with no history of cholecystectomy, accompanied by a normal-sized or slightly dilated common bile duct, suspicion for CGA should be raised. And there is a need to broaden diagnostic methods, and genetic sequencing and in-depth embryological studies may provide new insights into elucidating its etiology and improving diagnostic accuracy.

## Conclusion

Congenital gallbladder agenesis is a rare embryological anomaly of the biliary system, and its diagnosis and treatment are challenging. For patients with suspected atrophic cholecystitis, MRI should be performed. This can reduce the misdiagnosis rate of CGA, thereby avoiding unnecessary surgical interventions.

## Data Availability

The original contributions presented in this study are included in this article/supplementary material, further inquiries can be directed to the corresponding authors.
